# USP7 and VCP^FAF1^ define the SUMO/Ubiquitin landscape at the DNA replication fork

**DOI:** 10.1016/j.celrep.2021.109819

**Published:** 2021-10-12

**Authors:** André Franz, Pablo Valledor, Patricia Ubieto-Capella, Domenic Pilger, Antonio Galarreta, Vanesa Lafarga, Alejandro Fernández-Llorente, Guillermo de la Vega-Barranco, Fabian den Brave, Thorsten Hoppe, Oscar Fernandez-Capetillo, Emilio Lecona

**Affiliations:** 1 Institute for Genetics and Cologne Excellence Cluster on Cellular Stress Responses in Aging-Associated Diseases (CECAD), University of Cologne, 50931 Cologne, Germany; 2 Genomic Instability Group, Spanish National Cancer Research Centre (CNIO), Madrid 28029, Spain; 3 Center for Molecular Medicine Cologne (CMMC), University of Cologne, Cologne, Germany; 4 The Wellcome Trust and Cancer Research UK Gurdon Institute and Department of Biochemistry, University of Cambridge, Cambridge CB2 1QN, UK; 5 Institute of Biochemistry and Molecular Biology, University of Bonn, 53115 Bonn, Germany; 6 Science for Life Laboratory, Division of Genome Biology, Department of Medical Biochemistry and Biophysics, Karolinska Institute, 171 21 Stockholm, Sweden; 7 Chromatin, Cancer and the Ubiquitin System lab, Centre for Molecular Biology Severo Ochoa (CBMSO, CSIC-UAM), Department of Genome Dynamics and Function, Madrid 28049, Spain

**Keywords:** DNA replication, USP7, VCP, FAF1, ubiquitin, SUMO, DUB, MATH-33, CDC-48, UBXN-3

## Abstract

The AAA^+^ ATPase VCP regulates the extraction of SUMO and ubiquitin-modified DNA replication factors from chromatin. We have previously described that active DNA synthesis is associated with a SUMO-high/ubiquitin-low environment governed by the deubiquitylase USP7. Here, we unveil a functional cooperation between USP7 and VCP in DNA replication, which is conserved from *Caenorhabditis elegans* to mammals. The role of VCP in chromatin is defined by its cofactor FAF1, which facilitates the extraction of SUMOylated and ubiquitylated proteins that accumulate after the block of DNA replication in the absence of USP7. The inactivation of USP7 and FAF1 is synthetically lethal both in *C. elegans* and mammalian cells. In addition, USP7 and VCP inhibitors display synergistic toxicity supporting a functional link between deubiquitylation and extraction of chromatin-bound proteins. Our results suggest that USP7 and VCP^FAF1^ facilitate DNA replication by controlling the balance of SUMO/Ubiquitin-modified DNA replication factors on chromatin.

## Highlights

• VCP and USP7 control the SUMO/ubiquitin landscape during DNA replication
• VCP is recruited by FAF1 to chromatin-associated SUMO/ubiquitylated proteins
• FAF1 recognizes both SUMO and ubiquitin on target proteins
• The control of DNA replication by VCP^FAF1^ and USP7 is evolutionarily conserved

## Graphical abstract

**Figure:**
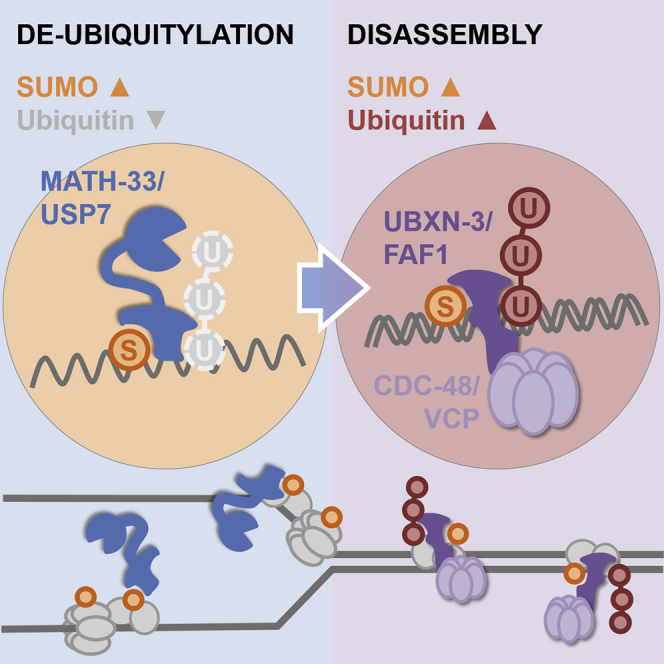

## Teaser

Chromatin at DNA replication forks is SUMO-rich and ubiquitin-poor. Franz et al. report that FAF1 is a dual reader of SUMO and ubiquitin targeting VCP to extract DNA replication factors from chromatin in *C. elegans* and human cells. Together with USP7, VCP^FAF1^ controls the SUMO/ubiquitin landscape during replication.

## Introduction

The duplication of genomic information requires an elaborated fine-tuning of multiple protein activities at the chromatin throughout DNA replication to ensure genome integrity (Burgers and Kunkel, 2017; Gaillard et al., 2015; Lecona and Fernández-Capetillo, 2014). There is growing evidence that protein SUMOylation and ubiquitylation controls the timely function of DNA replication factors by regulating their chromatin association (Abbas and Dutta, 2017; Keiten-Schmitz et al., 2020; Prudden et al., 2007; Rageul et al., 2019; Stelter and Ulrich, 2003; Wei and Zhao, 2017; Yates and Maréchal, 2018). For instance, after origin firing, the DNA clamp PCNA promotes the ubiquitylation, extraction, and degradation of specific factors to prevent the re-licensing at origins of replication (Arias and Walter, 2006; Coleman et al., 2015). In addition, SUMOylation can be used as a timer for the ubiquitylation and degradation of the Dbf4-dependet kinase (DDK) to control origin firing (Psakhye et al., 2019). During G1 MCM proteins are also SUMOylated, limiting their phosphorylation and the firing of new origins while during the elongation phase the SUMOylation of polymerase ε promotes DNA synthesis (Meng et al., 2019; Wei and Zhao, 2016).

On a global scale, proteomic analyses of the replisome revealed an overall higher concentration of SUMO compared to low levels of ubiquitin around active replication forks, suggesting that group SUMOylation of replication factors sustains efficient DNA replication (Dungrawala et al., 2015; Lopez-Contreras et al., 2013; Psakhye and Jentsch, 2012). In this context, the modification by SUMO could be restricting the ubiquitylation of replication factors by direct competition on the same lysine residues (Moldovan et al., 2007) or serve as a mark for the timely ubiquitylation of modified proteins by SUMO-targeted ubiquitin ligases (STUbLs) (Tatham et al., 2008; Uzunova et al., 2007). Along this line, we have recently described that the chromatin-bound SUMO-ubiquitin equilibrium is maintained by USP7, a de-ubiquitylating enzyme (DUB) that targets SUMO-modified factors to define the overall levels of SUMOylation and ubiquitylation at active DNA replication forks (Lecona and Fernandez-Capetillo, 2016; Lecona et al., 2016).

In chromatin the regulation of SUMO- and ubiquitin conjugated proteins often involves the valosin-containing protein (VCP, also known as cell-cycle defective protein 48 [CDC-48]) (Franz et al., 2014; Ye et al., 2017). CDC-48/VCP is a molecular segregase that liberates modified proteins from higher-order complexes, chromatin, or cellular membranes, thereby facilitating protein recycling, inactivation and/or degradation by the 26S proteasome (Bodnar and Rapoport, 2017; Cooney et al., 2019; Ndoja et al., 2014; Richly et al., 2005; Twomey et al., 2019). Substrate targeting of CDC-48/VCP is defined by its exclusive association with alternative cofactors (Buchberger et al., 2015; Hänzelmann and Schindelin, 2017). A prominent CDC-48/VCP cofactor is the heterodimer of ubiquitin-fusion degradation protein 1 (UFD-1/UFD1L) and nuclear protein localization protein 4 (NPL-4/NPLOC4), which serves as a versatile adaptor for CDC-48/VCP-mediated protein degradation at diverse cellular compartments including the endoplasmic reticulum (ER) (Braun et al., 2002), mitochondria (Mårtensson et al., 2019; Metzger et al., 2020), stalled ribosomes (Verma et al., 2013), or the nucleus (Franz et al., 2016; Khmelinskii et al., 2014). Interestingly, a complex of VCP and UFD1L:NPLOC4 together with the cofactor FAS-associated factor 1 (FAF1, VCP^UFD1L:NPLOC4:FAF1^ complex) has been shown to regulate the dynamic association of DNA replication factors with chromatin playing a role in origin licensing and the disassembly of the replisome (Franz et al., 2016; Sonneville et al., 2017).

Here, we identified the conserved and concerted function of USP7 and VCP in the control of DNA replication through unbiased genetic and proteomic approaches both in *C. elegans* and mammalian cells. We identified UBXN-3/FAF1 as a central cofactor for CDC-48/VCP in sensing SUMO- and ubiquitin modifications associated with DNA replication and counteracted by the DUB MATH-33/USP7. Together, our work demonstrates an intricate cooperation between USP7 and VCP^FAF1^ in the control of DNA replication fork progression by modulating the SUMO/ubiquitin landscape of chromatin-associated proteins.

## Results

### Genetic interaction between CDC-48/VCP and MATH-33/USP7

**Figure 1:**
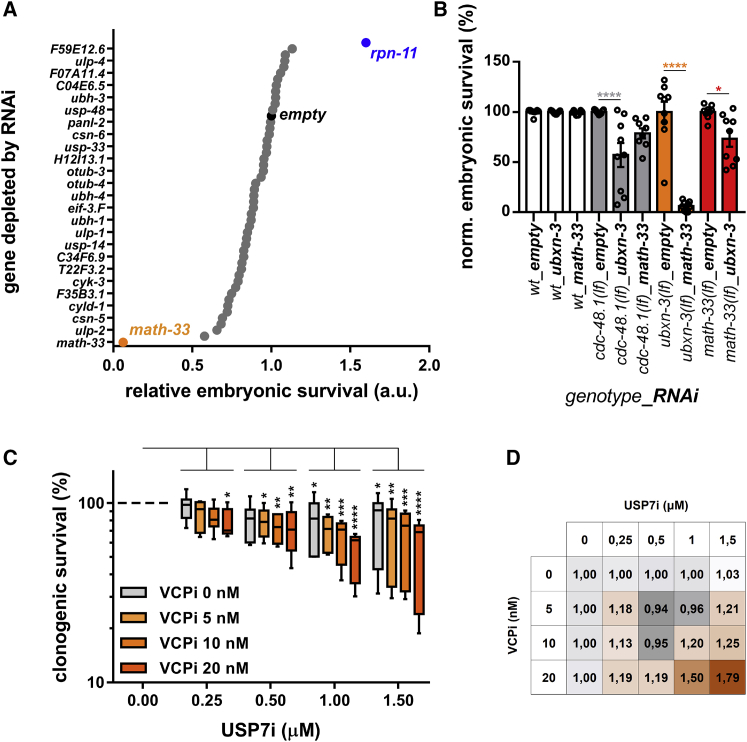
Conserved cooperation between MATH-33/USP7 and CDC-48/VCP^UBXN-3^ (A) Graph shows the embryonic survival of *ubxn-3(lf)* mutants relative to WT control when depleted for the genes encoding known or predicted DUBs. Both WT and *ubxn-3* were normalized to 100% embryonic survival for the *empty* control RNAi condition. The candidate screen was performed in two biological replicates. Graph shows the mean values. Strong genetic interaction was observed for *rpn-11*(RNAi) (blue) and *math-33*(RNAi) (orange). y axis displays every second RNA target that is plotted in the graph. (B) Graph shows normalized embryonic survival. The data validate synthetic embryonic lethality of *math-33*(RNAi) in *ubxn-3* mutants (orange bars) as well as in reverse genetic constellation (red bars) and in *cdc-48.1(lf)* mutants (gray bars). Circles indicate individual data points, bars show respective mean values, and error bars show standard error of the mean. Asterisks indicate statistical significance in one-way ANOVA Sidak’s multiple-comparison test. (C) The graph shows a whiskers plot (5–95^th^ percentile) of colony formation analysis of mESCs treated with indicated doses of USPi and VCPi alone or in combination. The data present three independent experiments, each performed in three technical replicates. Asterisks indicate statistical significance in two-way ANOVA Dunnett’s multiple-comparison test referring to the respective 0 μM USP7i condition. (D) Matrix shows observed colony formation defects upon combined USPi and VCPi treatments, relative to the expected additive effect of either single treatment. The higher the ratio (the darker the shade of orange), the stronger the observed synergy upon double-inhibition is. ^∗^p value < 0.05, ^∗∗^p value < 0.01, ^∗∗∗^p value < 0.001, ^∗∗∗∗^p value < 0.0001.

Our recent findings showed that CDC-48 regulates the association of DNA replication factors with chromatin in cooperation with its cofactors UFD-1, NPL-4, and UBXN-3 (CDC-48^UFD-1:NPL-4:UBXN-3^) (Mouysset et al., 2008; Franz et al., 2011, 2016). Since CDC-48 activity depends on substrate ubiquitylation, we asked whether de-ubiquitylation plays a regulatory role in this process. To address this question, we performed a candidate RNAi screen in *C. elegans* to deplete known and predicted DUBs in both the wild-type (WT) and the *ubxn-3(tm6658)* loss-of-function *(lf)* mutant. We monitored relative normalized embryonic survival and identified two DUBs, which specifically modulated embryonic lethality in the *ubxn-3(lf)* mutant (Figures 1A, S1A, and S1B). While the survival upon *rpn-11* depletion was increased in the *ubxn-3* mutants, reduced levels of *math-33* showed a remarkable synthetic lethality in this genetic background (Figure 1A). Follow-up experiments validated both the increased tolerance to *rpn-11* depletion as well as the strong synthetic lethality with *math-33* depletion in the *ubxn-3(lf)* mutant (Figure S1A). Although the genetic interaction between the proteasome subunit *rpn-11* and *ubxn-3* is of potential interest, the high embryonic lethality and meiotic defects associated with the depletion of *rpn-11* precluded a more detailed phenotypic analysis. Regarding MATH-33, we confirmed that the depletion of *ubxn-3* also reduced the survival in *math-33(lf)* mutant embryos (Figure 1B), supporting a non-directional genetic interaction between *ubxn-3* and *math-33*. Further, depletion of *math-33* in *cdc-48.1(lf)* mutants also resulted in decreased embryonic survival (Figure 1B), indicating that the synthetic lethality of *math-33(RNAi)* in the *ubxn-3(lf)* mutant is related to its function as a cofactor of CDC-48.

MATH-33 in *C. elegans* is the closest ortholog of mammalian USP7. Thus, we decided to explore whether the genetic interaction between MATH-33 and CDC-48 was conserved upon pharmacological inhibition of USP7 (USP7i) and VCP (VCPi). We treated mouse embryonic stem cells (mESCs) with increasing doses of USP7i and VCPi alone or combined and analyzed cell survival in a colony formation assay. Both USP7i and VCPi reduced the formation of colonies and the combination of both inhibitors led to a stronger decrease in colony formation (Figure 1C). The analysis of the combined inhibition of USP7 and VCP on cell survival revealed a synergistic interaction between both factors given the amplified rather than additive effect compared to single drug treatments (Figure 1D). Thus, pharmacological inhibition of USP7 and VCP in mESCs confirms the genetic interaction observed in *C. elegans*.

### MATH-33/USP7 cooperates with CDC-48/VCP and UBXN3/FAF1 in DNA replication

**Figure 2:**
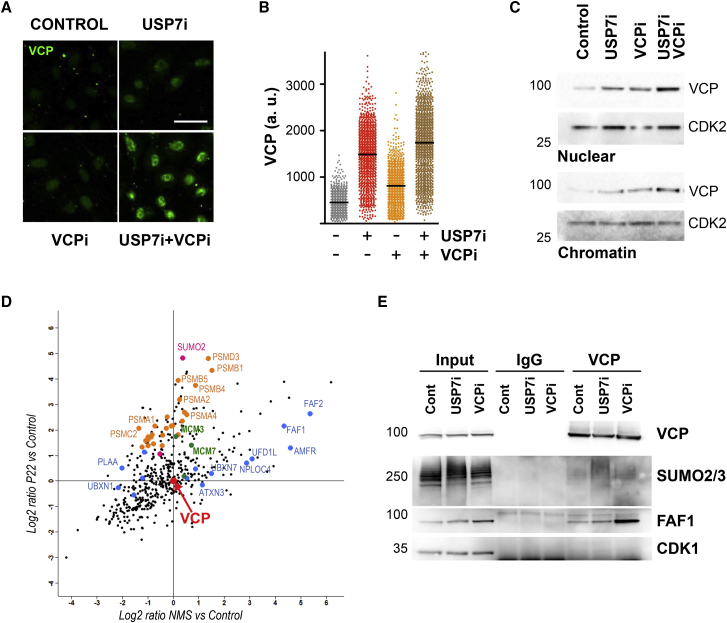
VCP accumulates on chromatin and interacts with FAF1 and SUMOylated proteins upon USP7 inhibition (A) Immunofluorescence of VCP in U2OS cells treated with DMSO (Control), 5 μM NMS873 (VCPi), 50 μM P22077 (USP7i), or a combination of both (USP7i+VCPi) for 4 h. Soluble, nuclear material was removed by pre-extraction previous to fixation. Scale bar, 50 μm. (B) Analysis of the levels of VCP on chromatin by high-throughput microscopy in U2OS cells treated with DMSO (C), 5 μM NMS873 (VCPi), 50 μM P22077 (USP7i), or a combination of both (USP7i-VCPi) for 4 h. (C) WB analysis of the levels of VCP in the soluble nuclear (Nuc) and chromatin (Chr) fractions obtained from HCT116 cells treated with DMSO (C), 5 μM NMS873 (VCPi), 50 μM P22077 (USP7i), or a combination of both (USP7i-VCPi) for 4 h. CDK2 is shown as a loading control. The experiments in (A)–(C) were repeated 3 times, and one representative experiment is shown. (D) Mass spectrometry analysis of the pull-down of VCP after cross-linking in HCT116 cells treated with 10 μM NMS873 or 50 μM P22077 for 4 h. The enrichment of proteins in each condition in two independent experiments was compared to DMSO-treated cells and normalized to the total amount of VCP in the samples. Enrichment upon VCPi treatment is shown on the x axis and enrichment upon USP7i treatment is shown on the y axis. SUMO2/3 is shown in pink, proteasome components in orange, components of the CMG helicase in green, and known VCP adaptors in blue. (E) WB analysis of the pull-down of VCP in cells treated as in (D). The levels of VCP, SUMO2/3, FAF1, and CDK1 were analyzed with specific antibodies. 5% of the input material is shown (Input). A control immunoprecipitation with a non-specific IgG was performed (IgG) and compared to the pull-down of VCP (VCP). The experiment was repeated three times with equivalent results, and an additional blot is shown in Figure S2.

Having established a genetic interaction between CDC-48 and MATH-33 in *C. elegans*, we aimed to gain insight into their functional connection. We recently described that USP7 inhibition results in the ubiquitylation of SUMOylated factors at the replisome leading to their accumulation in nuclear sub-domains that are distinct from PCNA foci (Lecona et al., 2016). Thus, we hypothesized that VCP might participate in the extraction of the SUMOylated and ubiquitylated proteins that accumulate on chromatin following USP7 inhibition. Supporting this view, high-throughput immunofluorescence experiments in pre-extracted nuclei of U2OS cells revealed that treatment with USP7i induced an accumulation of VCP on chromatin, which was exacerbated through the concomitant inhibition of VCP (Figures 2A, 2B, and S2A). This accumulation of VCP on chromatin was confirmed in cellular fractionation experiments (Figures 2C and S2B). Of note, and in agreement with our previous proteomic results (Lecona et al., 2016), we did not detect any changes in the ubiquitylation status or stability of VCP upon USP7 inhibition (Figures S2C and S2D), suggesting that the accumulation of VCP on chromatin is based on increased substrate interaction.

Next, we searched for substrates and cofactors that drive VCP to chromatin when USP7 is inhibited. We performed a proteomic analysis of the VCP interactome in whole cell extracts using the cross-linking agent dithiobis-succinimidyl propionate (DSP) to preserve the binding to its cofactors (Xue et al., 2016). Using this experimental setup, we immunoprecipitated VCP in control conditions as well as after treatment with USP7i or VCPi (Figure S2E). VCP cofactors were the most enriched proteins in this experiment (Figure S2F) and the changes induced by VCPi closely recapitulated results of a previous proteomics study (Xue et al., 2016) (Figure S2G). We compared the interactome of VCP in response to USP7i or VCPi (Figure 2D). Interestingly, SUMO2/3 was the most enriched VCP-interacting protein upon USP7i treatment (Figure 2D, pink dot), suggesting that VCP recognizes SUMOylated factors. We also identified a strong interaction of VCP with the proteasome induced by USP7 inhibition (Figure 2D, orange dots), although this interaction is unlikely to drive VCP to chromatin. In this regard, we detected increased binding of VCP with components of the CMG helicase upon USP7 inhibition (Figure 2D, green dots), linking VCP to the regulation of DNA replication under this condition. Focusing on the adaptors of VCP, the analysis identified FAF1 and FAF2 as the most enriched proteins that bind to VCP when treated with USP7i (Figure 2D, blue dots). The interaction of VCP with FAF1 and SUMOylated proteins upon USP7 inhibition was subsequently confirmed by immunoprecipitation experiments (Figures 2E, S2H, and S2I). These results suggest that CDC-48/VCP is relocated upon MATH-33/USP7 inhibition to extract replication factors that are SUMOylated. This observation is in agreement with our genetic screen suggesting that UBXN-3/FAF1 is the relevant adaptor for CDC-48/VCP in the context of limited MATH-33/USP7 activity.

**Figure 3:**
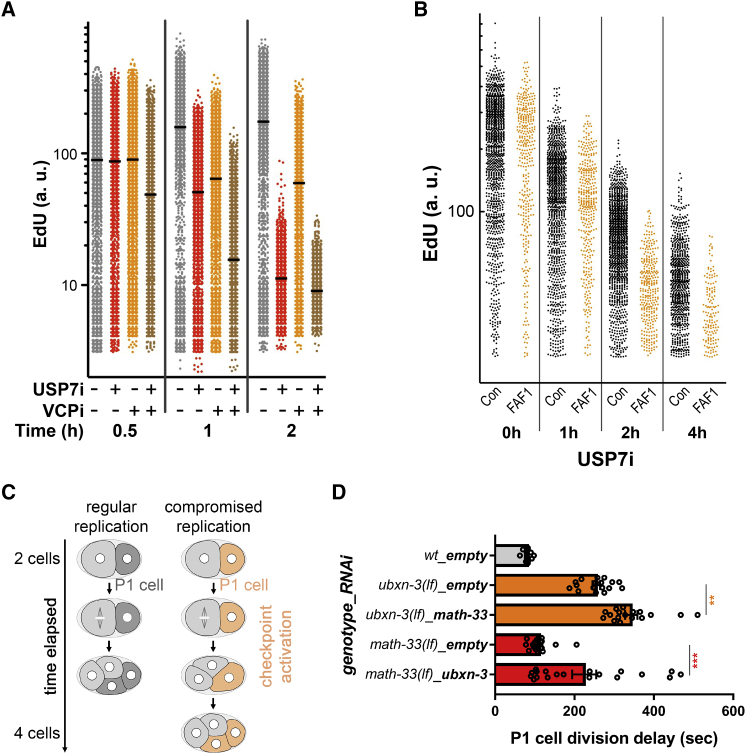
Cooperation between USP7/MATH-33 and VCP/CDC-48 in DNA replication (A) Analysis of EdU incorporation by immunofluorescence and high-throughput microscopy in HCT116 cells treated with DMSO, 5 μM NMS873 (VCPi), 50 μM P22077 (USP7i), or a combination of both for the indicated time. (B) RPE cells were transfected with a non-specific small interfering RNA (siRNA) (Con, black) or siRNA against FAF1 (FAF1, orange), and the levels of EdU incorporation were measured by high-throughput microscopy 48 h after transfection. Cells were treated with DMSO (Control) or 10 μM P22077 (USP7i) for the indicated times, and we show EdU positive cells. The experiment was repeated 3 times, and one representative result is shown. (C) Schematic representation of measurement of DNA replication checkpoint-mediated cell-cycle delay in *C. elegans* early embryos. (D) Graph shows synthetic effect of combined *ubxn-3* and *math-33* inactivation (orange and red bars) compared to the WT control (gray bar). Circles indicate individual data points, bars show mean values, and error bars show standard error of the mean. Asterisks indicate statistical significance in one-way ANOVA Sidak’s multiple-comparison test (^∗∗^p < 0.01, ^∗∗∗^p < 0.001).

We previously showed that USP7 inhibition arrests DNA replication and leads to the mislocalization of replication factors (Lecona et al., 2016). Given that we identified replication factors in the VCP interactome after USP7i, we decided to analyze whether USP7 and FAF1 cooperate in DNA replication. To this end, we synchronized RPE cells in G1/S with a double thymidine block and released them in the presence of VCPi. The inhibition of VCP during S phase led to a reduced EdU incorporation 6 h after release (Figure S3A). Although cells eventually completed DNA replication, the treatment with VCPi induced a block in G2/M (Magnaghi et al., 2013) and a gradual accumulation of VCP on chromatin together with its adaptors UFD1L, NPLOC4, and FAF1 (Figure S3B). In contrast to FAF1, FAF2 was depleted from chromatin upon VCP inhibition (Figure S3B). These data argue that, similar to recent findings in *C. elegans* (Franz et al., 2011, 2016), UBXN-3/FAF1, UFD-1/UFD1L, and NPL-4/NPLOC4 define the activity of CDC-48/VCP during DNA replication. In this sense, previous reports have shown that these three adaptors interact simultaneously with CDC-48/VCP (Ewens et al., 2014; Hänzelmann et al., 2011; Lee et al., 2013; Sasagawa et al., 2010). In line with the synergistic effects of USP7i and VCPi in reducing cell viability, the combination of both agents showed a strong effect in the inhibition of DNA replication, reducing the incorporation of EdU further than any of the inhibitors used alone (Figure 3A). This effect was confirmed using two additional but structurally unrelated USP7 inhibitors (Figures S3C and S3D) and in two additional cell lines (Figures S3E and S3F). Similar to the inhibition of VCP, the depletion of FAF1 but no other adaptors (Figures S4A and S4B) enhanced the reduction of EdU incorporation induced by USP7i (Figures 3B, S4C, and S4D), confirming that USP7 cooperates with FAF1 in the regulation of DNA replication.

As an orthogonal approach to these studies on DNA replication, we measured the timing of cell division in *C. elegans* embryos. A delay in the division of the P1 cell serves as an established readout for impaired DNA replication and the activation of the DNA-replication checkpoint kinases ATL-1/ATR and CHK-1/CHK1 (Brauchle et al., 2003; Encalada et al., 2000; Franz et al., 2011; Mouysset et al., 2008) (Figure 3C). Consistent with our data in mammalian cell culture, both the silencing of *math-33* in *ubxn-3(lf)* mutants as well as the depletion of *ubxn-3* in *math-33(lf)* mutants significantly delayed cell-division timing compared to the mutants alone, supporting a coordinated activity of CDC-48^UBXN-3^ and MATH-33 in ensuring faithful DNA replication (Figure 3D). Together, these results indicate that CDC-48/VCP^UBXN3/FAF1^ has a conserved function in the regulation of DNA replication in coordination with MATH-33/USP7.

### Role of CDC-48/VCP^UBXN-3/FAF1^ in the control of SUMOylated/ubiquitylated proteins at chromatin

**Figure 4:**
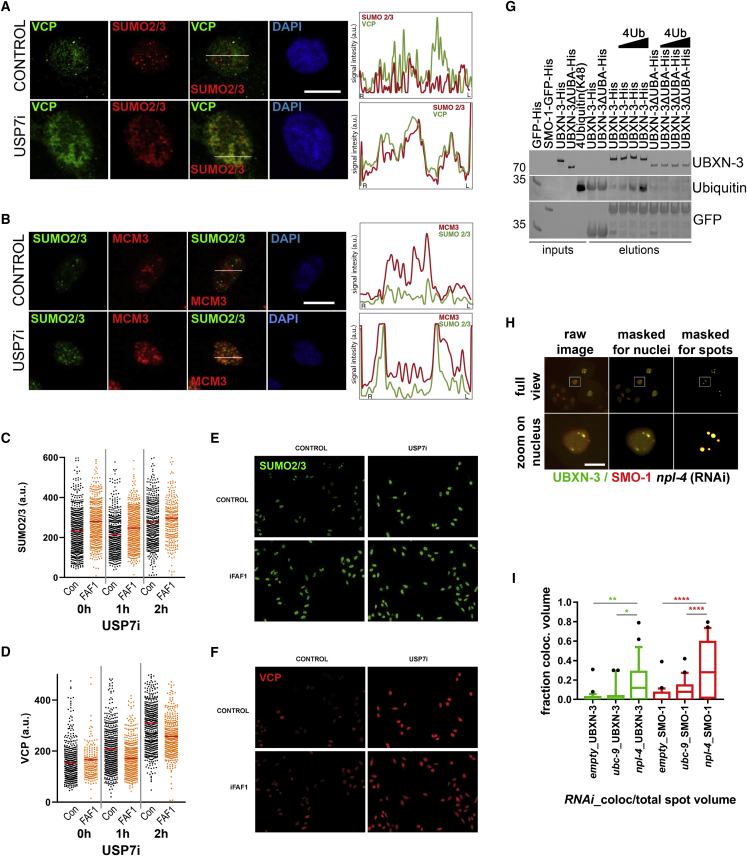
USP7 and VCP^FAF1^ functions in DNA replication converge into the SUMOylation pathway (A and B) Immunofluorescence analysis of chromatin-bound VCP (green) and SUMO2/3 (red) (A) or SUMO2/3 (green) and MCM3 (red). (B) Levels in U2OS cells that were either untreated (CONTROL) or after treatment with 50 μM USP7i for 4 h. DNA was stained with DAPI (blue). The overlay for the different staining is also shown. Scale bar, 10 μm. The intensity of the individual stainings was quantified along the line indicated in the figure to assess the co-localization of the analyzed proteins (right). (C and D) HeLa cells were transfected with a non-specific siRNA (Con, black) or siRNA against FAF1 (FAF1, orange), and the levels of SUMO2/3 (C) or VCP (D) on chromatin were measured by high-throughput microscopy 48 h after transfection. Cells were treated with DMSO (Control) or 25 μM P22077 (USP7i) for the indicated times. (E and F) Representative images taken at 4 h of treatment are shown. The experiment was repeated 3 times, and one representative result is shown. (G) Western blot analysis of the inputs and eluates of an *in vitro* pull-down using GFP and SMO-1-GFP as bait. UBXN-3 or a deletion variant of the N-terminal UBA (ΔUBA) domain were used along with increasing amounts of tetra-Ubiquitin chains linked via Lysine 48 (4Ub(K48)). Note, that detection of Ubiquitin revealed cross-reactivity of the antibody with GFP. SMO-1-GFP-bound UBXN-3 can efficiently recover 4Ub(K48) without affecting UBXN-3 binding to SMO-1. UBXN-3(ΔUBA) interacts with SMO-1-GFP, while it is deficient in ubiquitin binding. (H) Schematic representation of quantitative analysis of UBXN-3 (green) and SMO-1 (red) spots in *C. elegans* embryonic nuclei. Raw images were masked for embryonic tissue and nuclei, before nuclear spots were defined for subsequent analysis. Images show a representative of npl-4-depleted embryos, immune labeled with UBXN-3 and SMO-1 antibodies. (I) Quantification of co-localized UBXN-3 (green) and SMO-1 (red) spot volume in embryos depleted for indicated gene products relative to green or red total spot volume, respectively. Whisker plots of the 10–90^th^ percentile are shown, and statistical significance was interrogated using one-way ANOVA Sidak’s multiple-comparison test and is indicated by asterisks (^∗^p < 0.05, ^∗∗^p < 0.01, ^∗∗∗∗^p < 0.0001). Number of independent images analyzed: n_*empty*__(RNAi)_ = 25, n_*ubc-9*__(RNAi)_ = 34, n_*npl-4*__(RNAi)_ = 38. Scale bars, 10 μm in (A) and (B) and 5 μm in (H).

We have previously shown that USP7 controls DNA replication by maintaining the equilibrium between SUMO and ubiquitin modification at the replication fork (Lecona et al., 2016; Lecona and Fernandez-Capetillo 2016). Since our proteomic data show that VCP interacts with SUMOylated proteins and replication factors after USP7i, we decided to further explore their localization by immunofluorescence. Consistent with several proteomic studies that did not find an enrichment of VCP in replicating versus mature chromatin (Alabert et al., 2014; Dungrawala et al., 2015; Lopez-Contreras et al., 2013), VCP shows a punctate pattern throughout the nucleus with a partial overlap with PCNA or SUMO2/3 in non-perturbed conditions (Figures 4A and S4E). In contrast, we detected VCP at the chromatin in large regions where SUMOylated proteins accumulated in response to USP7i (Figures 4A and S4E), and these regions occupied by VCP and SUMO did not overlap with PCNA foci (Figures 4A and S4E). A similar increase in SUMO-rich domains upon USP7 inhibition was also seen for replication factors such as POLD2 (Figure S4F). In agreement with the increased interaction of VCP with MCM proteins after USP7i, MCM3 was also localized to the SUMO-rich patches after treatment with USP7i (Figure 4B).

Given that VCP binds its substrates via ubiquitin and SUMO conjugates, we wondered about the impact of these modifications on the chromatin localization of VCP. To this end, we treated cells with specific ubiquitylation and SUMOylation inhibitors (MLN7243 and ML792, respectively) (Hyer et al., 2018; Magnaghi et al., 2013) and detected reduced binding of VCP to chromatin only when both modifications were removed in U2OS cells (Figure S4G). In contrast, the increase in chromatin-bound VCP mediated by USP7i was strongly reduced upon treatment with the ubiquitylation inhibitor (Figure S4H). Of note, defective ubiquitylation also limited the accumulation of SUMOylated factors on chromatin (Figure S4J) after USP7 inhibition, while it does not affect SUMOylation in control conditions (Figure S4I), suggesting that the combination of both modifiers determines chromatin recruitment of VCP. Accordingly, the inhibition of SUMO-conjugation reduced the amount of chromatin-bound VCP induced by USP7i and further enhanced the effect of ubiquitin inhibition on VCP accumulation (Figure S4H). These data indicate that the accumulation of ubiquitylated and SUMOylated proteins triggered by USP7i fosters chromatin binding of VCP, which is governed by FAF1.

FAF1 has been previously shown to control the extraction of ubiquitylated proteins from chromatin to facilitate proteasomal degradation. However, it remained unclear whether it also targets SUMOylated proteins. The depletion of FAF1 led to increased levels of SUMOylated factors on chromatin in control conditions and enhanced the accumulation of SUMOylated proteins upon USP7i (Figures 4C, 4E, and S4K). Concomitantly, FAF1 depletion reduced the accumulation of VCP on chromatin after USP7 inhibition (Figures 4D, 4F, and S4L), indicating that FAF1 targets VCP for the extraction of SUMOylated proteins from chromatin. Indeed, FAF1 immunoprecipitation confirmed its interaction with SUMOylated proteins and showed that USP7 inhibition enhances the binding of FAF1 and SUMO-modified proteins (Figure S4M). As expected from previous reports, FAF1 also associates with ubiquitylated proteins and this interaction is further increased upon USP7 inhibition (Figure S4M). Finally, we checked the binding of FAF1 to DNA replication factors. FAF1 pull-down co-purified POLD2, one of the proteins that is relocated upon USP7 inhibition, while it did not bind RPA2 or histone H2A (Figures S4F and S4M). To further define the link between SUMO/ubiquitin and UBXN-3/FAF1, we went back to the *C. elegans* model system. We carried out *in vitro* pull-down experiments that showed a direct binding of UBXN-3 to SUMO (SMO-1 in *C. elegans*). A SMO-1 variant that is defective in interaction with SUMO-interaction-motifs (SIM) (SMO-1^F29A_V31A(∗∗)^-GFP) (Jardin et al., 2015; Xie et al., 2010) revealed a markedly decreased binding to UBXN-3, underscoring a direct SMO-1 to SIM interaction (Figure S5A). Since point mutations affecting the predicted SIMs (Zhao et al., 2014) in the N- and C-termini of UBXN-3 were not conclusive, we decided to use truncated variants of UBXN-3 to map the region responsible for the interaction with SMO-1. Deletion of the protein region corresponding to the UBL domains in FAF1 completely abolished the binding between UBXN-3 and SMO-1 (Figure S5B). In contrast, the deletion of the UBA domain that mediates the binding of UBXN-3 to ubiquitin (Franz et al., 2016; Song et al., 2009) did not affect the interaction with SMO-1 (Figure S5B). These data recapitulated the *in vitro* protein-protein interaction of FAF1 and SUMO that has been reported in mammals (Wang et al., 2019) and suggest that UBXN-3 includes conserved binding domains for both SUMO and ubiquitin. To further support this conclusion, we immunoprecipitated UBXN-3 with SMO-1-GFP in the presence of increasing concentrations of tetra-Ub(K48) chains. A SMO-1/UBXN-3 complex could effectively bind to tetra-Ub(K48) chains, suggesting that UBXN-3 is able to bind SUMO and ubiquitin simultaneously. In contrast, GFP-SMO-1 precipitated equivalent amount of UBXN-3 lacking the UBA domain, but this mutant no longer interacted with ubiquitin (Figure 4G). These results indicate that UBXN-3/FAF1 provides independent domains for simultaneous binding of ubiquitin and SUMO-modified factors to recruit CDC-48/VCP to chromatin during DNA replication. Finally, we assessed the functional relevance of SUMOylation for CDC-48/VCP-dependent DNA replication. To this end, we monitored the appearance of collapsed replication forks and followed increased replication stress by measuring the formation of RAD-51 foci (Ackermann et al., 2016; Hashimoto et al., 2011; Petermann et al., 2010). The inactivation of the CDC-48^UFD-1:NPL-4^ complex using *npl-4*(RNAi) caused an accumulation of RAD-51 foci (Figures S5C and S5D), as has been documented previously (Franz et al., 2011, 2016; Mouysset et al., 2008). Interestingly, the depletion of the SUMO-conjugating enzyme *ubc-9*, which does not induce RAD-51 foci formation, aggravated replication fork collapse induced by *npl-4*(RNAi) both in WT and *math-33(lf)* worms (Figures S5C and S5D), which suggests that SUMOylation is particularly important when CDC-48/VCP activity is compromised. Besides increased replication fork collapse, our previous results have shown that the depletion of CDC-48^UFD-1:NPL-4^ correlates with the formation of UBXN-3 nuclear foci (Figure S5E) (Franz et al., 2016). Quantitative analysis of nuclear spots revealed increased UBXN-3 spot volume (Figure S5F) as well as amplified volume shared by both UBXN-3 and SMO-1 foci upon *npl-4* (RNAi) (Figures 4H and 4I). These findings confirm our conclusion that UBXN-3 processes SUMO conjugates, which is enforced when CDC-48^UFD-1:NPL-4^ activity is affected. Together, our data provide evidence for a functional cooperation between VCP^FAF1^/CDC-48^UBXN-3^ and USP7/MATH-33, which is critical for a balanced SUMO/ubiquitin equilibrium and replication fork progression.

## Discussion

The presence of a SUMO-high and ubiquitin-low environment in active replication forks suggests that the assembly of DNA replication factories might follow the same model of “group-SUMOylation” that was previously proposed for DNA repair (Psakhye and Jentsch, 2012). The extent of SUMOylation and ubiquitylation in the replisome is kept in check by the deubiquitylase USP7 (Lecona et al., 2016). Here, we show that the role of USP7 is coordinated with VCP by recognizing SUMOylated and ubiquitylated proteins via its cofactor FAF1. In yeast, Cdc48/VCP targets SUMOylated factors through SUMO interacting motifs (SIMs) in Cdc48 and Ufd1, but these SIMs are not conserved in mammals (Bergink et al., 2013; Nie et al., 2012). Instead, we have confirmed the direct interaction between UBXN-3/FAF1 and SUMO that was recently reported in mammalian cells (Wang et al., 2019). Our data suggest that UBXN-3/FAF1 interacts simultaneously with ubiquitylated proteins via the N-terminal UBA domain (Franz et al., 2016; Song et al., 2005) and with SUMOylated proteins through the adjacent protein region corresponding to the UBL domains of human FAF1. Further, we show that UBXN-3/FAF1 works as part of a VCP^UFD1L:NPLOC4:FAF1^ complex during DNA replication, which is in agreement with previous analysis showing that FAF1 interacts with VCP only in the context of a VCP^UFD1L:NPLOC4^ complex (Bodnar et al., 2018; Hänzelmann et al., 2011). While VCP^UFD1L:NPLOC4^ complexes are known to work in several cellular processes, our data suggest that FAF1 specifically directs this complex on chromatin during DNA replication. Thus, the depletion of NPLOC4 might not be able to efficiently deplete the VCP^UFD1L:NPLOC4:FAF1^ complex and affect DNA replication as FAF1 knockdown does.

Based on our results, we propose that the VCP cofactor FAF1 acts as a sensor for SUMO and ubiquitin modifications during DNA replication, which might display a dual role in cooperation with USP7 during this process. First, FAF1 might coordinate the extraction of SUMOylated and ubiquitylated proteins to limit their accumulation on active replication forks. We hypothesize that an excessive accumulation of SUMO and ubiquitin-modified replication factors could promote the premature ubiquitylation of the replisome. As a second layer of control, USP7 limits the excessive ubiquitylation of replication factors establishing a two-step control process to ensure the replisome is not prematurely evicted. Second, upon efficient ubiquitylation of replication factors FAF1 would target VCP^UFD1L:NPLOC4^ for the timely extraction from chromatin, as demonstrated for CDT-1 and the CMG complex (Franz et al., 2011, 2016; Sonneville et al., 2017). Our data define the functional cooperation between VCP and USP7 to control the chromatin-associated SUMO and ubiquitin landscape. We provide evidence that ubiquitin and SUMO modifications at the replisome are interdependent, since the inhibition of USP7 triggers increased SUMOylation, which is enhanced by a concomitant increase in ubiquitylation. Thus, SUMO and ubiquitin function as additive, intermolecular tagging systems for the coordinated eviction of DNA replication factors. It remains to be determined how SUMOylation of the replisome is regulated and whether it provides additional functions during DNA replication. Moreover, our data support the idea that USP7 activity is regulated in time and space to coordinate modification of replication factors with DNA replication fork progression.

Our findings provide mechanistic insights into the role of ubiquitin and SUMO in control of DNA replication. We propose an interdependent coordination of ubiquitylation and SUMOylation events at the replisome mediated by USP7, which facilitates the spatiotemporal extraction and degradation of chromatin-associated DNA replication factors by VCP^FAF1^ to support faithful DNA replication.

## STAR★Methods

### Key resources table

**Table:** 

Reagent or resource	Source	Identifier
**Antibodies**		

rabbit polyclonal UBXN-3	Biogenes	animal 22717 and 22718, reference: Franz et al., 2016
mouse monoclonal SMO-1	Developmental Studies Hybridoma Banks	clone 6F2, RRID: AB_2618393, reference: Pelisch et al., 2014
mouse monoclonal Ubiquitin	Upstate, Sigma Aldrich	clone P4D1-A11 (Cat#05-944); RRID:AB_441944
mouse monoclonal anti-living colors (GFP)	Clontech	clone JL-8 (Cat#632381); RRID:AB_2313808
rabbit polyclonal RAD-51	Novus Biologicals	Cat#29480002, Animal# SDQ0811; RRID:AB_2284913
rabbit polyclonal USP7	Bethyl	A300-033A; RRID:AB_203276
rabbit polyclonal VCP	Bethyl	A300-589A; RRID:AB_495512
mouse monoclonal SUMO2/3	Developmental Studies Hybridoma Banks	Clone 8A2; RRID:AB_2198421
mouse monoclonal SUMO2/3	MBL	M114-3; RRID:AB_592769
rabbit polyclonal MCM3	Juan Mendez lab (CNIO)	
rabbit polyclonal FAF1	Bethyl	A302-810A; RRID:AB_10633846
mouse monoclonal FAF1	Novus Biologicals	H00011124-B01P; RRID:AB_1261301
mouse monoclonal FAF2	Santa Cruz Biotechnology	sc-374098; RRID:AB_10918565
rabbit polyclonal ASPSCR1	Novus Biologicals	NBP1-90079; RRID:AB_11039773
rabbit polyclonal NPLOC4	Novus Biologicals	NBP1-82166; RRID:AB_11006469
rabbit polyclonal UFD1L	Abcam	ab155003
rabbit polyclonal POLD1	Santa Cruz Biotechnology	sc-10784; RRID:AB_2166441
rabbit polyclonal POLD2	Bethyl	A304-322A; RRID:AB_2620518
rabbit polyclonal PCNA	Santa Cruz Biotechnology	sc-56; RRID:AB_628110
mouse monoclonal p53	Santa Cruz Biotechnology	sc-126 clone DO-1; RRID:AB_628082
mouse monoclonal CDK2	Santa Cruz Biotechnology	sc-163; RRID:AB_631215
mouse monoclonal H2A	Cell Signaling	3636; RRID:AB_2118801
mouse monoclonal VCP	Abcam	Ab11433; RRID:AB_298039
Goat Anti-Rabbit IgG (H+L), HRP	ThermoFisher	Cat#31460; RRID:AB_228341
Goat Anti-Mouse IgG (H+L), HRP	ThermoFisher	Cat#31430; RRID:AB_228307
Alexa Fluor 488 anti-mouse	ThermoFisher	Cat#A11001; RRID:AB_2534069
Alexa Fluor 488 anti-rabbit	ThermoFisher	Cat#A21441; RRID:AB_2535859
Alexa Fluor 594 anti-mouse	ThermoFisher	Cat#A11005; RRID:AB_141372
Alexa Fluor 647 anti-rabbit	ThermoFisher	Cat#A21443; RRID:AB_1500685
donkey anti mouse 680	Li-Cor	Cat#926-32222; RRID:AB_621844
donkey anti rabbit 800	Li-Cor	Cat#926-32213; RRID:AB_621848
GFP-Trap, magnetic agarose	Chromotek	Cat#gtma; RRID:AB_2631358

**Chemicals, peptides, and recombinant proteins**		

CB-5083	Insight Biotechnology	Cas#1542705-92-9
NMS-873	Tocris	Cat#6180
P22077	bio techne	Cat#4485
P22077	Merck-Millipore	Cat#662142
MLN7243	Chemietek	Cat#CT-M7243
ML792	Synthetized in the CNIO	N/A
BAY 11-7082	Santa Cruz Biotechnology	Sc-200615
FT681	MedChemExpress	HY-107985
Human Tetra-Ubiquitin (K48-linked)	BostonBiochem	Cat#UC-210B

**Deposited data**		

Protein interaction IP-MS data	This paper, PRIDE	PXD018623

**Experimental models: Cell lines**		

Mouse embryonc stem cells (mESC)	isolated as described in Reference	Balmus et al., 2019
Human: HCT116	ATCC	CCL-247
Human: U2OS	ATCC	HBT-96
Human: RPE	ATCC	CRL-4000
Human: HeLa	ATCC	CCL-2
Human: MCF-7	ATCC	HTB-22

**Experimental models: Organisms/strains**		

*C. elegans* Strain FX544: *cdc-48.1(tm544)II*	Caenorhabditis Genetics Center (CGC)	WormBase ID: WBStrain00007563
*C. elegans* Strain FX6658: *ubxn-3(tm6658)II*	National Bioresource Project (NBRP)	N/A
*C. elegans Strain* FX6724: *math-33(tm6724)V*	Caenorhabditis Genetics Center (CGC)	WormBase ID: WBVar02125553
Bacterial RNAi feeding libraries Ahringer or ORFeomeWS112 libraries	Geneservice Ltd, available via Source BioScience	Laboratories of Julie Ahringer, Marc Vidal

**Oligonucleotides**		

siRNA smartpool human FAF1	Dharmacon (Horizon Discovery)	L-009106-00-0005
siRNA smartpool human FAF2	Dharmacon (Horizon Discovery)	L-010649-02-0005
siRNA smartpool human ASPSCR1	Dharmacon (Horizon Discovery)	L-006489-02-0005
siRNA smartpool human NPLOC4	Dharmacon (Horizon Discovery)	L-020796-01-0005

**Recombinant DNA**		

Plasmid: GFP::His	this paper	N/A
Plasmid: SMO-1::GFP::His	this paper	N/A
Plasmid: SMO-1^∗∗^::GFP::His	this paper	N/A
Plasmids: UBXN-3::His and truncation variants UBXN-3Δ4-87, UBXN-3Δ4-281, UBXN-3Δ279-440	Franz et al., 2016	N/A
Plasmid: pCL-His-hUbi	Young et al., 2011	N/A

**Software and algorithms**		

ImageJ (FIJI)	Schindelin et al., 2012	https://imagej.net/Downloads
Adobe Photoshop Elements 14	Adobe	N/A
Prism 7	GraphPad	N/A
Imaris	Oxford Instruments	N/A
MaxQuant	Max-Planck Institute of Biochemistry	N/A
FLOWJO	FlowJo, LCC	N/A

### Resource availability

#### Lead contact

Further information and requests for resources should be directed to and will be fulfilled by the Lead Contact, Emilio Lecona (elecona@cbm.csic.es).

#### Materials availability

All reagents generated in this study are available upon request to the Lead Contact and upon signature of the corresponding Material Transfer Agreement, if necessary.

### Experimental model and subject details

#### Cell lines

HCT116 Human Colon Carcinoma, Male

U2OS Human Bone Osteosarcoma, Female

RPE Human Retinal Pigment Epithelial Cells, Female

MCF-7 Human Breast Adenocarcinoma, Female

HeLa Human Cervical Carcinoma, Female

These cells were grown in DMEM with 10% FBS, penicillin (100 IU/ml), streptomycin (100 mg/ml) and glutamine (300 mg/ml). For passaging, cells were washed once with warm PBS and trypsinized with Trypsin-EDTA (Sigma). Trypsin was inactivated by the addition of fresh media and the cell suspension was centrifuged at 400 *g* for 3 min.

#### Murine embryonic stem cells

WT mESCs were obtained from Atm ± oocytes (see Key Resources Table), cultured in DMEM (PAN-biotech) supplemented with 15% FBS (Thermo Fisher Scientific), 1x penicillin-streptomycin-glutamine (Thermo Fisher Scientific), 1x MEM Non-essential amino acids (100x, Thermo Fisher Scientific), 1x Sodium Pyruvate (100x, Thermo Fisher Scientific), 60 Mio units of recombinant mouse leukemia inhibitory factor (LIF) protein (Merck) and 0.8% of 2-Mercapthoethanol (Sigma, Merck). For ESCs, tissue culture flasks/plates were coated with 0.1% gelatin solution for at least 15 min at RT prior to cell seeding. For passaging, cells were washed once with warm PBS and trypsinized with Trypsin-EDTA (Sigma). Trypsin was inactivated by the addition of fresh media and the cell suspension was centrifuged at 400 *g* for 3 min. After resuspension in fresh media, cells were counted by using a Countess II (Thermo Fisher Scientific) according to manufacturer’s protocol.

#### C. elegans

*C. elegans* nematodes were treated according to standard protocols at 20°C, unless otherwise stated (Brenner, 1974). The Bristol strain N2 was used as wild-type. Mutants used in this study are *ubxn-3(tm6658)II, cdc-48.1(tm544)II,* and *math-33(tm6724)V*.

### Method details

#### Extract preparation, transfections and treatments

P22077 (Merck-Millipore), NMS873 (Tocris), cycloheximide (Sigma, Merck), ML792 (Synthetized in the CNIO) and MLN7243 (Chemietek) were dissolved in DMSO; cells were incubated for the indicated time in the presence of the inhibitor or an equivalent amount of DMSO. Whole cell extracts were prepared by lysing cells in 50 mM Tris, pH 7.5, 8 M Urea, and 1% Chaps. Cytosolic and nuclear extracts were prepared following the protocol described before (Lecona et al., 2008) and the chromatin fraction was then extracted in 50 mM Tris, pH 7.5, 8 M Urea, and 1% Chaps. Transfection of RPE cells with specific siRNA was carried out using Lipofectamine RNAimax (Invitrogen, Thermo Fisher Scientific) according to the manufacturer’s instructions and using pools of 4 specific siRNA directed against the indicated proteins (Dharmacon, Horizon Discovery). Transfection of HCT116 cells with the pCL-His-hUbi plasmid (Young et al., 2011) was carried out using Lipofectamine 2000 (Invitrogen, Thermo Fisher Scientific) following the manufacturer’s instructions.

#### Colony formation assay

The day before drug treatment was started, cells were seeded in a 6-well plate at a concentration of 250 cells per well. The following day, cells were treated with respective concentrations of USPi (P22077, Bio Techne) and/or VCPi (CB-5083, Insight Biotechnology) drugs for 5 to 7 days, consequently cells were washed gently once with PBS, fixed with methanol for 20 min and stained with crystal violet (20% methanol (v/v), 0.25% crystal violet (w/v) in water). Finally, the plates were scanned, and the number of colonies were automatically calculated using the Fiji software. Specifically, thresholds were set accordingly to identify colonies and the ‘analyze particles’ tool was used to count colonies automatically. All data was normalized to the non-treated control samples to compensate for seeding differences and/or seeding efficiency. Samples treated with combinations of USP7i and VCPi were normalized to the VCP inhibitor mono treatment/treatment only to account for toxicity derived from VCP inhibition.

#### Cell synchronization

RPE cells were synchronized using a double thymidine block. Cells were incubated in the presence of 1 mM Thymidine for 16h at 37°C. Then, cells were washed once in PBS and released in DMEM for 8h at 37°C. The culture medium was replaced with DMEM containing 1mM Thymidine and cells were incubated for 16h at 37°C. Again, cells were washed once and released in DMEM.

#### Antibodies

For mammalian cells the antibodies against USP7 (Bethyl, A300-033A), VCP (Bethyl, A300-589A), SUMO2/3 (MBL, M114-3 and Developmental Studies Hybridoma Banks, clone 8A2), MCM3 (Rabbit polyclonal antibody provided by Juan Méndez), FAF1 (Bethyl, A302-810A and Novus Biologicals, H00011124-B01P), FAF2 (Santa Cruz Biotechnology, sc-374098), ASPSCR1 (Novus Biologicals, NBP1-90079), NPLOC4 (Novus Biologicals, NBP1-82166), UFD1L (Abcam, ab155003), POLD1 (Santa Cruz Biotechnology, sc-10784), POLD2 (Bethyl, A304-322A), PCNA (Santa Cruz, sc-56), p53 (Santa Cruz Biotechnology, DO-1, sc-126), CDK2 (Santa Cruz Biotechnology, M2, sc-163), H3S10P (Millipore, # 06-570), H2A (Cell Signaling, #3636) were used for Western Blot and immunofluorescence. VCP antibody (Abcam, ab11433) was used for immunoprecipitation. In *C. elegans* studies we used primary antibodies used in immune-histochemistry are rabbit anti-UBXN-3 (selfmade in cooperation with Biogenes), mouse anti-SMO-1 (Developmental Studies Hybridoma Banks, 6F2), rabbit anti-RAD-51 (Novus Biologicals (29480002)). Primary antibodies used for western blotting are mouse anti-GFP (Clonetech, JL-8), anti-UBXN-3. Fluorophore-conjugated secondary antibodies where obtained from Thermo Fisher Scientific or Li-Cor, respectively.

#### Cross-linking with DSP

The immunoprecipitation of VCP after cross-linking was carried out following the protocol in Xue et al. (Xue et al., 2016). Briefly, cells were treated with the cross-linking agent Dithiobis[succinimidyl propionate] (DSP) (Thermo Fisher Scientific, Waltham, MA), freshly prepared as a 200 mM stock solution in dimethyl sulfoxide (DMSO) and diluted to 0.8 mM in PBS. Cells were washed twice with PBS and incubated with DSP for 20 min at room temperature. DSP was replaced with 25 mM Tris-HCl (pH 7.4) and cells were incubated for 10 min at room temperature to quench the reaction. Then, cells were scraped in ice-cold PBS and stored at −80°C. Cells were resuspended in buffer A (25 mM Tris-HCl pH 7.4, 150 mM NaCl, 1 mM EDTA, 5% glycerol, 1% Nonidet P-40) and lysed by sonication at 4°C. Lysates were cleared by centrifugation at 16,000 g for 5 min.

#### Immunoprecipitation

500 μg of protein were diluted at 1 mg/ml in 50 mM Tris pH 7.9, 200 mM NaCl (BC200) and centrifuged for 10 min at 20,000 g at 4°C. Protein G Dynabeads (Invitrogen, Thermo Fisher Scientific) were washed twice in BC200 and then incubated with anti-VCP antibody or a non-specific IgG in the presence of 0.5 mg/ml BSA in BC200. Loaded Dynabeads were washed 5 times in BC200 and incubated with the cleared supernatant ON at 4°C. The beads were washed five times with BC200 with 0.05% IGEPAL CA630 (Sigma, Merck). One fourth of the beads were eluted in loading buffer and the rest was processed by the Proteomic Unit in the CNIO.

#### Sample preparation for proteomic analysis

Proteins were eluted from the magnetic beads in two consecutive steps by shaking for 45 min at 1400 rpm in an Eppendorf Thermomixer in 2 bead volumes (aprox 100 μl) of elution buffer (8 M Urea, 15 mM TCEP, 100 mM Tris-HCl pH = 8.0). The beads were separated using a magnetic stand. The supernatant obtained was digested by means of standard FASP protocol. Briefly, proteins were alkylated (50 mM CAA, 20 min in the dark, RT) and sequentially digested with Lys-C (Wako) (protein:enzyme ratio 1:50, o/n at RT) and trypsin (Promega) (protein:enzyme ratio 1:100, 6 h at 37°C). Resulting peptides were desalted using C_18_ stage-tips.

#### Mass spectrometry

LC-MS/MS was done by coupling a nanoLC-Ultra 1D+ system (Eksigent) to a LTQ Orbitrap Velos mass spectrometer (Thermo Fisher Scientific) via a Nanospray Flex source (Thermo Fisher Scientific). Peptides were loaded onto a reversed-phase ReproSil Pur C18-Aq 5 μm 0.3 × 10 mm trapping cartridge (SGE Analytical), and washed for 10 min at 2.5 μL/min with loading buffer (0.1% FA). The peptides were eluted from a RP ReproSil Pur C18-AQ 1.9 μm 400 × 0.075 mm home-made column by application of a binary gradient consisting of 4% ACN in 0.1% FA (buffer A) and 100% ACN in 0.1% FA (buffer B), with a flow rate of 250 nL/min. Peptides were separated using the following gradient: 0 to 2 min 2%–6% B, 2 to 90 min 6%–20% B, 90 to 103 min 20%–35% B, 103 to 113.5 min 35%–98% B and 103.5 to 113.5 min 98%B. The peptides were electrosprayed (1.8 kV) into the mass spectrometer with a PicoTip emitter (360/20 Tube OD/ID μm, tip ID 10 μm) (New Objective), a heated capillary temperature of 325°C and S-Lens RF level of 60%. The mass spectrometer was operated in a data-dependent mode, with an automatic switch between MS and MS/MS scans using a top 15 method (threshold signal ≥ 800 counts and dynamic exclusion of 60 s). MS spectra (350-1500 m/z) were acquired in the Orbitrap with a resolution of 60,000 FWHM (400 m/z). Peptides were isolated using a 1.5 Th window and fragmented using collision induced dissociation (CID) with linear ion trap read out at a NCE of 35% (0.25 Q-value and 10 ms activation time). The ion target values were 1E6 for MS (500 ms max injection time) and 5000 for MS/MS (100 ms max injection time).

#### Mass spectrometry-data analysis

Raw files were processed with MaxQuant (v 1.5.3.30) using the standard settings against a human protein database (UniProtKB/Swiss-Prot, December 2013, 20,187 sequences) supplemented with contaminants. Carbamidomethylation of cysteines was set as a fixed modification whereas oxidation of methionines and protein N-term acetylation as variable modifications. Minimal peptide length was set to 7 amino acids and a maximum of two tryptic missed-cleavages were allowed. Results were filtered at 0.01 FDR (peptide and protein level). Afterward, the “proteinGroup.txt” file was loaded in Perseus (v1.5.5.2) for further statistical analysis. LFQ values were normalized using the VCP protein levels, except for IgG controls. Missing values were imputed from the observed normal distribution of intensities. A Welc's t test with a permutation-based FDR was performed comparing each condition (P22 treatment, NMS treatment and not treated control) versus the IgG controls and only proteins with a q-value < 0.05 and a log2 ratio higher than 2 were considered as potential interactors. Only interactors with a log2 ratio > 1.5 or < −1.5 for the P22 and NMS treated samples versus not treated samples were considered as regulated.

#### Purification of ubiquitylated proteins

1 mg of protein from the chromatin fraction was diluted in 50 mM Tris pH 7.9, 8 M urea. 100 μL Ni-NTA resin (QIAGEN) was equilibrated in the same buffer and incubated with the extract rotating for 1h at room temperature. The column was washed with 3 mL 50 mM Tris pH 7.9, 8 M urea and proteins were eluted by heating the resin in loading buffer for 10’ at 70°C.

#### Fluorescence microscopy and high throughput microscopy

For immunofluorescence of chromatin bound proteins, cells were seeded on 0.1% gelatin, then the soluble material was pre-extracted with CSKI buffer for 4-6 minutes (10 mM Pipes, pH 6.8, 100 mM NaCl, 300 mM sucrose, 3 mM MgCl_2_, 1 mM EGTA, and 0.5% Triton X-100) before fixation in mSTF buffer (150 mM 2-Bromo-2-nitro-1,3-propanediol, 108 mM diazolidinyl urea, 10 mM Na Citrate, 50 mM EDTA (pH 5.7)). Cells were permeabilized in 100 mM Tris-HCl (pH 7.4), 50 mM EDTA (pH 8.0), 0.5% Trion X-100 followed by the staining for specific proteins using standard protocols.

For high throughput microscopy, cells were grown on μCLEAR bottom 96-well plates (Greiner Bio-One) and immunofluorescence was performed using standard procedures. Analysis of DNA Replication by EdU incorporation was done using Click-It (Invitrogen, Thermo Fisher Scientific) following manufacturers’ instructions.

In all cases, images were automatically acquired from each well using an Opera High-Content Screening System (Perkin Elmer). A 20x magnification lens was used and images were taken at non-saturating conditions. Images were segmented using DAPI signals to generate masks matching cell nuclei from which the mean signals for the rest of the stainings were calculated. Data were represented with the use of the Prism software (GraphPad Software).

#### Flow cytometry

For the analysis of the cell cycle, cells were incubated with 20 μM EdU for 30 minutes. Then, cells were trypsin-digested, washed with cold PBS once and fixed in 4% PFA or in CSKI buffer. After permeabilization with 0.25% Triton, the EdU was labeled by a Click reaction and the DNA was stained with DAPI 0.5 μg/ml in the presence of 0.25 mg/ml RNase A. All samples were analyzed in a BD LSRFortessa or in a FACSCanto II cell analyzer. The results were analyzed using the FlowJo software (FlowJo, LLC).

#### *C. elegans* RNAi-mediated gene depletion

RNAi-mediated depletion was achieved using the feeding method (Kamath et al., 2001). Bacteria inducibly expressing double-stranded (ds)RNA of respective target genes were taken from the Ahringer or ORFeomeWS112 libraries (Geneservice Ltd, available via Source BioScience). The *empty* feeding vector was used as control. Bacteria were grown in liquid culture over-night, diluted to an optical density (OD)_600_ of 0,1 the following day and grown to an OD_600_ of 1. dsRNA expression was induced by adding IPTG to a final concentration of 2mM for 30-60 minutes shaking at 37°C. Bacteria were then seeded onto growth media containing 2 mM IPTG at stored at room temperature (RT). For double-depletion experiments dsRNA induction was performed over-night shaking at RT, before bacteria were concentrated by centrifugation by a factor of five before seeding on IPTG containing growth media. Eggs harvested from gravid adult worms using alkaline hypochlorite solution were seeded onto growth plates seeded with RNAi bacteria and incubated at 20°C until worm reached adult stage for experimental analysis. To determine embryonic lethality, six gravid adults per data point where transferred to fresh RNAi plates and allowed to lay eggs for five to six hr. Then worms where removed from the plates and eggs where incubated at 20°C over-night. The following day the larvae hatched as well as the unhatched eggs where counted to calculate the penetrance of embryonic lethality. To facilitate better comparison embryonic survival individual data points of the untreated condition were normalized to the cumulative average of all experiments. The *ubxn-3(lf)* mutants displays an embryonic lethality of approximately 30% in untreated conditions.

#### *C. elegans* microscopy and image acquisition

For time-lapse microscopy, embryos were extruded from gravid hermaphrodites with the help of injection needles, transferred onto 3% agar pads in M9 buffer before microscopic analysis. An AxioImager.M1 or Z1 microscope equipped with an AxioCam 503mono camera (Carl Zeiss) was used for image acquisition. Time-lapse recordings in 10 s intervals were acquired until embryos completed the four-cell stage. Timing of cell division was estimated as described previously (Brauchle et al., 2003, Encalada et al., 2000). The same AxioImager.M1 or Z1 microscopes were also used for epifluorescence image acquisition. Confocal images were acquired using the Yokogawa CSU-X1 spinning disc module mounted to a Nikon TiE microscope stand, operated by Volocity software (Perkin Elmer). The spinning disc microscope is maintained and provided in the CECAD imaging facility. Z stacks were recorded with 200 nm distances between optical sections and projected into one single image using the maximum intensity projection in FIJI software. For the quantitative analysis images were processed and analyzed using Imaris software (version 9.5.1, Bitplane AG, Switzerland). In brief, individual channels were masked for embryonic tissue and baseline subtracted, followed by masking for nuclei and red/green spots by manual thresholding. To allow comparison of parameters in control and RNAi-depleted samples spots were defined for all sample types, albeit UBXN-3 spots were only obviously visible after *npl-4*(RNAi), as shown previously.

#### *C. elegans* immunotechniques

Immunostaining of early embryos was done essentially according to the ‘freeze-crack’ protocol. Gravid worms were dissected onto poly-lysine-coated slides (Thermo Fisher Scientific) and frozen in liquid nitrogen, followed by incubation in methanol at −20°C for 20 min and in acetone at −20°C for 20 min. After rehydration in PBS and blocking in 5% BSA, embryos were incubated with primary antibody overnight at 4°C. Incubation with the fluorescently labeled secondary antibodies (Life Technologies) was done at room temperature for 1 hr. Embryos were then mounted in DAPI Fluoromount G medium (SouthernBiotech). For quantification of RAD-51 positive embryos, all embryos on a slide where counted, irrespective of developmental stage, by focusing through embryos to categorize into RAD-51 foci positive or negative. For western blotting, purified proteins and worm lysates were separated by SDS-PAGE and transferred to nitrocellulose membranes (Whatman, Protran). Membranes were blocked in 3% milk solution and incubated with the primary antibodies overnight at 4°C in RotiBlock (Carl Roth). Incubation with fluorescently labeled secondary antibodies was done at room temperature, before detection of signals using the Li-Cor Odyssey scanner.

#### *In vitro* binding studies

Recombinant proteins where expressed in *Escherichia coli* strain BL21 Codon Plus (DE3) RIL (Agilent). Expression of proteins was induced by IPTG supplementation over night at 18°C. After Lysozyme treatment cells were lysed by sonication (Bandelin Sonopuls) and His-tagged proteins where precipitated using Ni-NTA resin (QIAGEN). Proteins where eluted using Imidazole, which was subsequently removed from the buffer by gelfiltration. Protein concentrations where determined using the NanoDrop 8000 (Thermo Fisher Scientific) and snap frozen in liquid nitrogen. Binding studies where performed at RT. Briefly, GFP-fusion proteins where bound to GFP-Trap (Chromotek) and incubated with identical amounts (m/m, usually 10 μg)) of UBXN-3-His for 2 hr. After washing, proteins where eluted from magnetic beads at 95°C for 5 min using 2x Laemmli buffer. For simultaneous binding to tetra-Ubiquitin-chains, increasing amounts of 4Ub(K48) were added to the reaction after 1hr incubation time and incubated for another 1hr, followed by subsequent elution. Total amounts of 4UB(K48) used were 0, 3, 6, and 12 μg, respectively.

### Quantification and statistical analysis

The details for the methods, quantification and statistical analysis can be found in the figure legends except for the proteomics analysis that can be found in the STAR Methods. Additional information of the acquisition and processing of the data can be found in the STAR Methods.

One-way Anova Sidak’s multiple comparison test was used to analyze embryonic survival, cell cycle delay and co-localization in immunofluorescence studies in *C. elegans*.

Colony formation assays were evaluated in Two-way Anova Dunnett’s multiple comparison test referred to the respective 0 μM USP7i condition.

Paired t test was used to analyze western blot and high-throughput microscopy experiments in human cell lines.

In proteomic studies a Welc's t test with a permutation-based FDR was performed comparing each condition (P22 treatment, NMS treatment and not treated control) versus the IgG controls and only proteins with a q-value < 0.05 and a log2 ratio higher than 2 were considered as potential interactors.

## Data Availability

The mass spectrometry data has been deposited in the PRIDE repository (Project accession PXD018623). This paper does not report original code. Any additional information required to reanalyze the data reported in this paper is available from the lead contact upon request.

## Supplemental information

Document S1. Figures S1–S6

Document S2. Article plus supplemental information
