# Supplementary material for: USP7 and VCPFAF1 define the SUMO/Ubiquitin landscape at the DNA replication fork
Source: Cell Rep. 2021 Oct 12;37(2):109819. doi: 10.1016/j.celrep.2021.109819 (PMC8527565; doi:10.1016/j.celrep.2021.109819)
Supplement: Document S1. Figures S1–S6 [file mmc1.pdf]

**Cell Reports, Volume *37***

## Supplemental information

# USP7 and VCP$^{FAF1}$ define the SUMO/Ubiquitin landscape at the DNA replication fork

**André Franz, Pablo Valledor, Patricia Ubieto-Capella, Domenic Pilger, Antonio Galarreta, Vanesa Lafarga, Alejandro Fernández-Llorente, Guillermo de la Vega-Barranco, Fabian den Brave, Thorsten Hoppe, Oscar Fernandez-Capetillo, and Emilio Lecona**

# Supplementary Figure 1

**A**

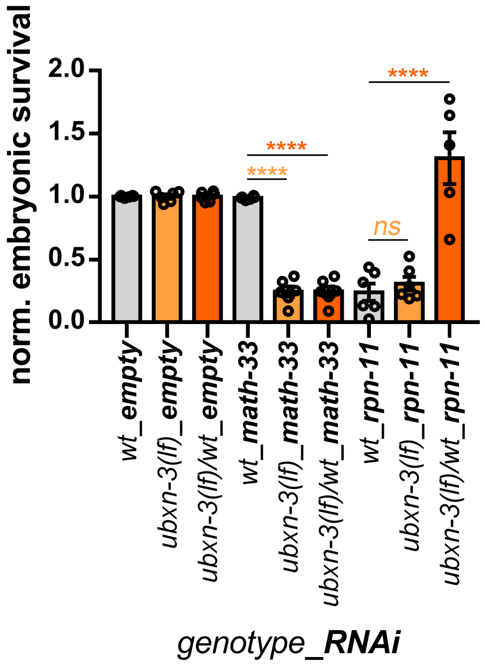


**B**

| RNAi target_# | locus | gene | mean embryonic survival *ubxn-3(lf)* / wt |
|---|---|---|---|
| 1 | H19N07.2 | *math-33* | 0,06 |
| 2 | Y40G12A.2 | *ubh-2* | 0,58 |
| 3 | Y38A8.3 | *ulp-2* | 0,65 |
| 4 | E01B7.1 | *usp-50* | 0,68 |
| 5 | B0547.1 | *csn-5* | 0,69 |
| 6 | F36D4.5 | *F36D4.5* | 0,72 |
| 7 | F40F12.5 | *cyld-1* | 0,72 |
| 8 | T24B8.7 | *T24B8.7* | 0,73 |
| 9 | F35B3.1 | *F35B3.1* | 0,75 |
| 10 | H34C03.2 | *H34C03.2* | 0,77 |
| 11 | ZK328.1 | *cyk-3* | 0,79 |
| 12 | F28F8.6 | *atx-3* | 0,80 |
| 13 | T22F3.2 | *T22F3.2* | 0,81 |
| 14 | R10E11.3 | *usp-46* | 0,81 |
| 15 | C34F6.9 | *C34F6.9* | 0,82 |
| 16 | Y71H2AR.3 | *josd-1* | 0,82 |
| 17 | C13B4.2 | *usp-14* | 0,84 |
| 18 | K08B4.5 | *K08B4.5* | 0,84 |
| 19 | T10F2.3 | *ulp-1* | 0,85 |
| 20 | T05H10.1 | *T05H10.1* | 0,86 |
| 21 | F46E10.8 | *ubh-1* | 0,87 |
| 22 | Y48A5A.2 | *ulp-3* | 0,87 |
| 23 | D2013.7 | *eif-3.F* | 0,89 |
| 24 | K02C4.3 | *K02C4.3* | 0,89 |
| 25 | C08B11.7 | *ubh-4* | 0,89 |
| 26 | Y50C1A.1 | *otub-2* | 0,89 |
| 27 | B0546.2 | *otub-4* | 0,90 |
| 28 | Y71A12B.9 | *usp-3* | 0,93 |
| 29 | F21D5.2 | *otub-3* | 0,95 |
| 30 | B0035.3 | *B0035.3* | 0,95 |
| 31 | H12I13.1 | *H12I13.1* | 0,95 |
| 32 | F37A4.5 | *F37A4.5* | 0,97 |
| 33 | K09A9.4 | *usp-33* | 0,97 |
| 34 | T27A3.2 | *usp-5* | 0,97 |
| 35 | Y67H2A.6 | *csn-6* | 0,99 |
| 36 | F38B7.5 | *duo-1* | 0,99 |
| 37 | F31E3.4 | *panl-2* | 1,00 |
| 38 | empty | *empty* | 1,00 |
| 39 | F30A10.10 | *usp-48* | 1,01 |
| 40 | C41D11.2 | *eif-3.H* | 1,02 |
| 41 | Y40G12A.1 | *ubh-3* | 1,03 |
| 42 | C25D7.8 | *otub-1* | 1,03 |
| 43 | C04E6.5 | *C04E6.5* | 1,03 |
| 44 | Y67D2.2 | *Y67D2.2* | 1,04 |
| 45 | F07A11.4 | *F07A11.4* | 1,06 |
| 46 | Y106G6H.12 | *duo-3* | 1,08 |
| 47 | C41C4.6 | *ulp-4* | 1,09 |
| 48 | F29C4.5 | *duo-2* | 1,09 |
| 49 | F59E12.6 | *F59E12.6* | 1,13 |
| 50 | K07D4.3 | *rpn-11* | 1,60 |
| 51 | C50C3.6 | *prp-8* | dev. compromissed |
| 52 | R12E2.3 | *rpn-8* | dev. compromissed |
| 53 | F09D1.1 | *F09D1.1* | dev. compromissed |
| 54 | K02F2.4 | *ulp-5* | dev. compromissed |
| 55 | T06E6.1 | *T06E6.2* | dev. compromissed |

**Supplementary Figure 1. Validation of genetic interaction of *rpn-11(RNAi)* and *math-33(RNAi)* in *ubxn-3(lf)* mutants.** Related to Figure 1 (A) Graph shows normalized embryonic survival in wt (grey bars) and *ubxn-3(lf)* mutants (light orange bars). The respective relative embryonic survival *ubxn-3(lf/wt)* is shown in dark orange. Circles display individual data points, whereas bars show the mean values, error bars show standard error of the mean. Asterisks indicate statistical significance in One-way Anova Sidak's multiple comparison test. (**** p-value <0,0001, ns not significant). (B) Table summarizes mean relative embryonic survival wt/ubxn-3(lf) when depleted for indicated RNAi targets. Data is plotted in Figure 1A. Genes resulting in developmental phenotypes when depleted by RNAi are indicated as dev. compromised and were consequently censored from the embryonic survival measurements.

# Supplementary Figure 2

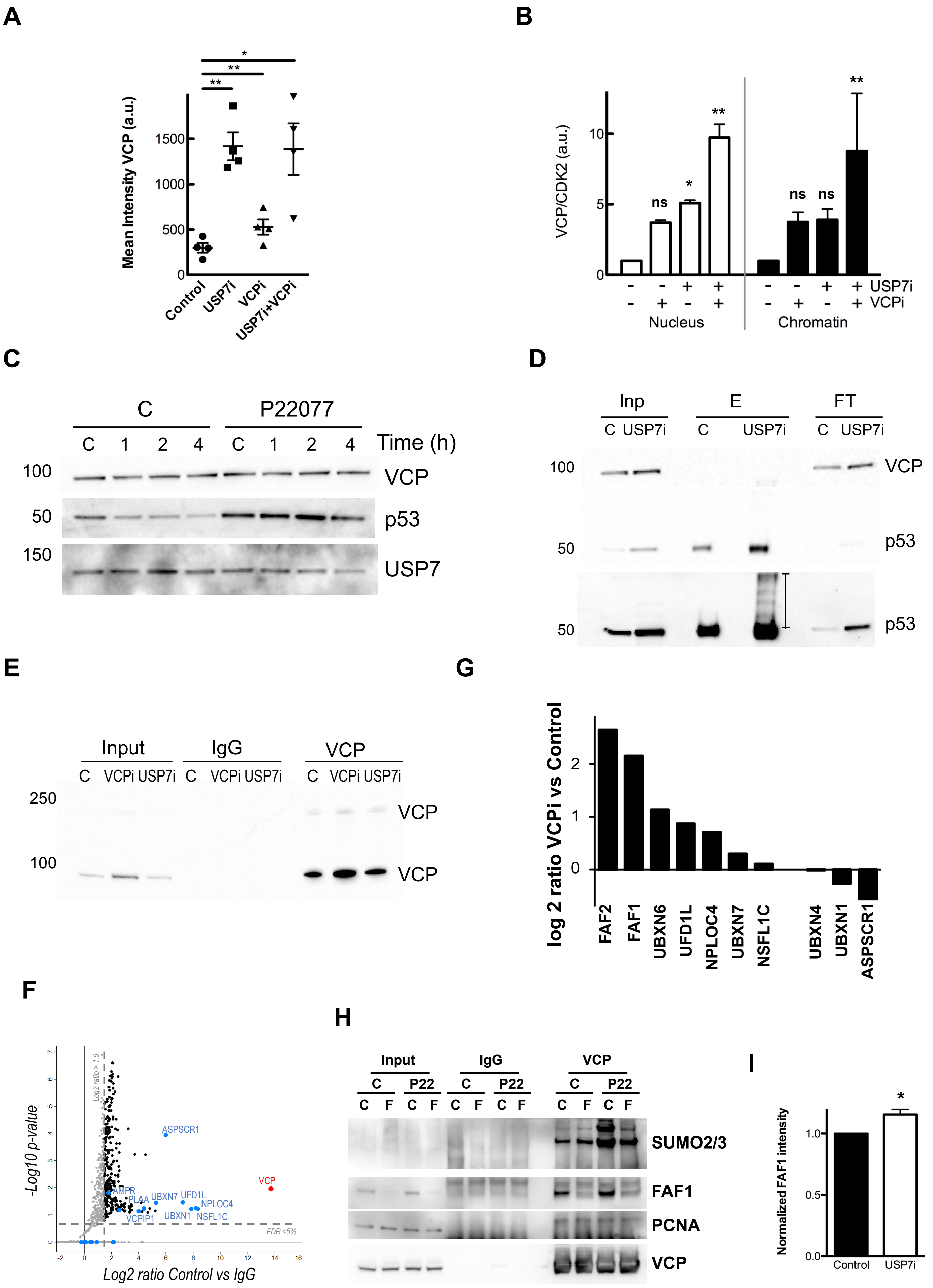

**Supplementary Figure 2. Stability and interactome of VCP after USP7 and VCP inhibition.** Related to Figure 2. (A) Quantification of the mean intensity of VCP on chromatin across four independent experiments as in Figure 2A-B. (B) Quantification of the levels of VCP in the soluble nuclear and chromatin fractions from 2 independent experiments as in Figure 2C. The levels of VCP were normalized to the presence of CDK2. Statistical significance was analyzed using a paired t-test. *$p<0.05$; **$p<0.01$ (C) WB analysis of VCP, p53 and USP7 in whole cell extracts from HCT116 cells treated with DMSO (C) or 50 μM P22077 (USP7i) and cycloheximide for the indicated times. One representative experiment out of three is shown. (D) HCT116 cells were transfected with a pCL-His-ubiquitin construct. 48 hrs after transfection cells were treated with DMSO as a control (C) or 50 μM P22077 (USP7i) for 4 hrs. Ubiquitylated proteins were pulled-down using a Ni-NTA resin and analyzed by WB with antibodies specific for VCP, p53 and p21. 5% of the input material is shown (Inp). The elution (E) and the flow-through (FT) fractions of control and USP7i treated cells are shown. The experiment was repeated twice with similar results. (E) WB analysis of the immunoprecipitation of VCP after cross-linking in HCT116 cells treated with DMSO (C) 10 μM NMS873 (VCPi) or 50 μM P22077 (USP7i) for 4hrs. 1% of the input material is shown together with the pull-down using a non-specific IgG or an antibody specific for VCP. The upper band is due to incomplete reversion of the cross-link with DSP. (F) Mass spectrometry analysis of the proteins in the pull-down of VCP. The enrichment in VCP pull-down compared to the immunoprecipitation with a non-specific IgG is plotted versus the p-value of the enrichment. (G) Enrichment of known adaptors in the interaction with VCP after treatment with 10 μM NMS873 (VCPi) for 4hrs compared to samples from DMSO

treatment. (H) Additional WB analysis of the pull down of VCP in cells treated as in Figure 2D-E. The levels of SUMO2/3, FAF1, PCNA and VCP were analyzed with specific antibodies. 5% of the input material is shown (Input). A control immunoprecipitation with a non-specific IgG was performed (IgG) and compared to the pull-down of VCP (VCP). As an additional control the pull down of VCP IP carried out in cells transfected with an siRNA against FAF1 (F) and compared to a non-specific siRNA (C). (I) Quantification of the levels of FAF1 in the VCP pull-down from 3 independent experiments. Statistical significance was analyzed using a paired t-test. $*p<0.05$.

## Supplementary Figure 3

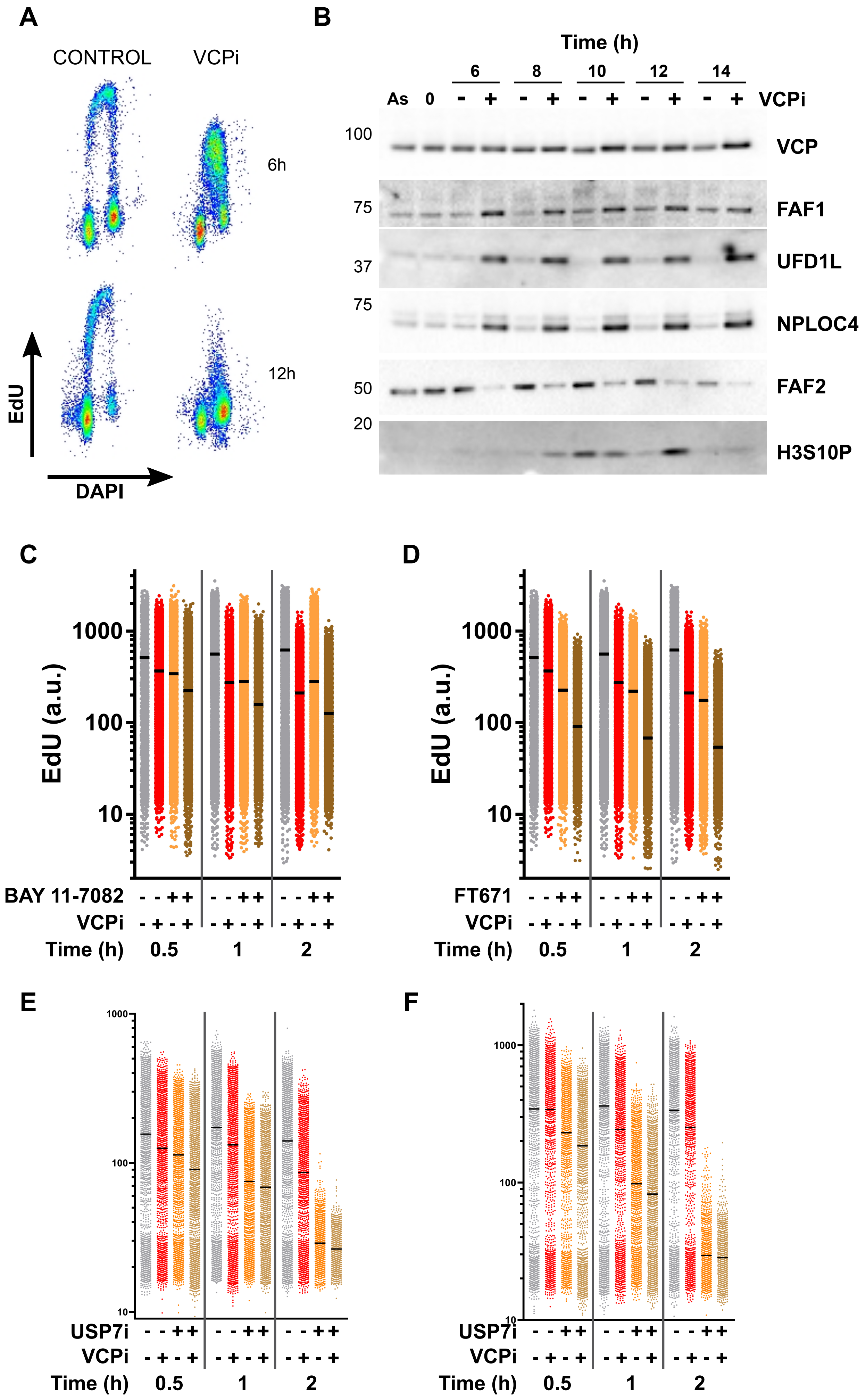

**Supplementary Figure 3. Regulation of DNA replication by VCP and USP7.** Related to Figure 3. (A) RPE cells were synchronized in G1/S using a double thymidine block and released in the presence of DMSO (control) or 10 μM NMS873 (VCPi). Cell cycle was analyzed 6 h and 12 h after release, measuring DNA content with DAPI and monitoring S phase through EdU incorporation. (B) WB analysis of the chromatin fraction of RPE cells synchronized in G1/S and released in the presence of DMSO or 10 μM NMS873. Extracts from asynchronous RPE cells (As) and cells before release (0) are shown as a control. The experiments in (A-B) were carried out three times with similar results. (C-D) Analysis of EdU incorporation by immunofluorescence and high throughput microscopy in HCT116 cells treated with DMSO, 5 μM NMS873 (VCPi), 17.5 μM BAY 11-7082 (C) or 20 μM FT671 (D) or a combination of both for the indicated time points. (E-F) Analysis of EdU incorporation by immunofluorescence and high throughput microscopy in MCF7 (E) and HeLa (F) cells treated with DMSO, 5 μM NMS873 (VCPi), 25 μM P22077 (USP7i) or a combination of both for the indicated time. The experiments in (C-F) were repeated three times and one representative result is shown.

# Supplementary Figure 4

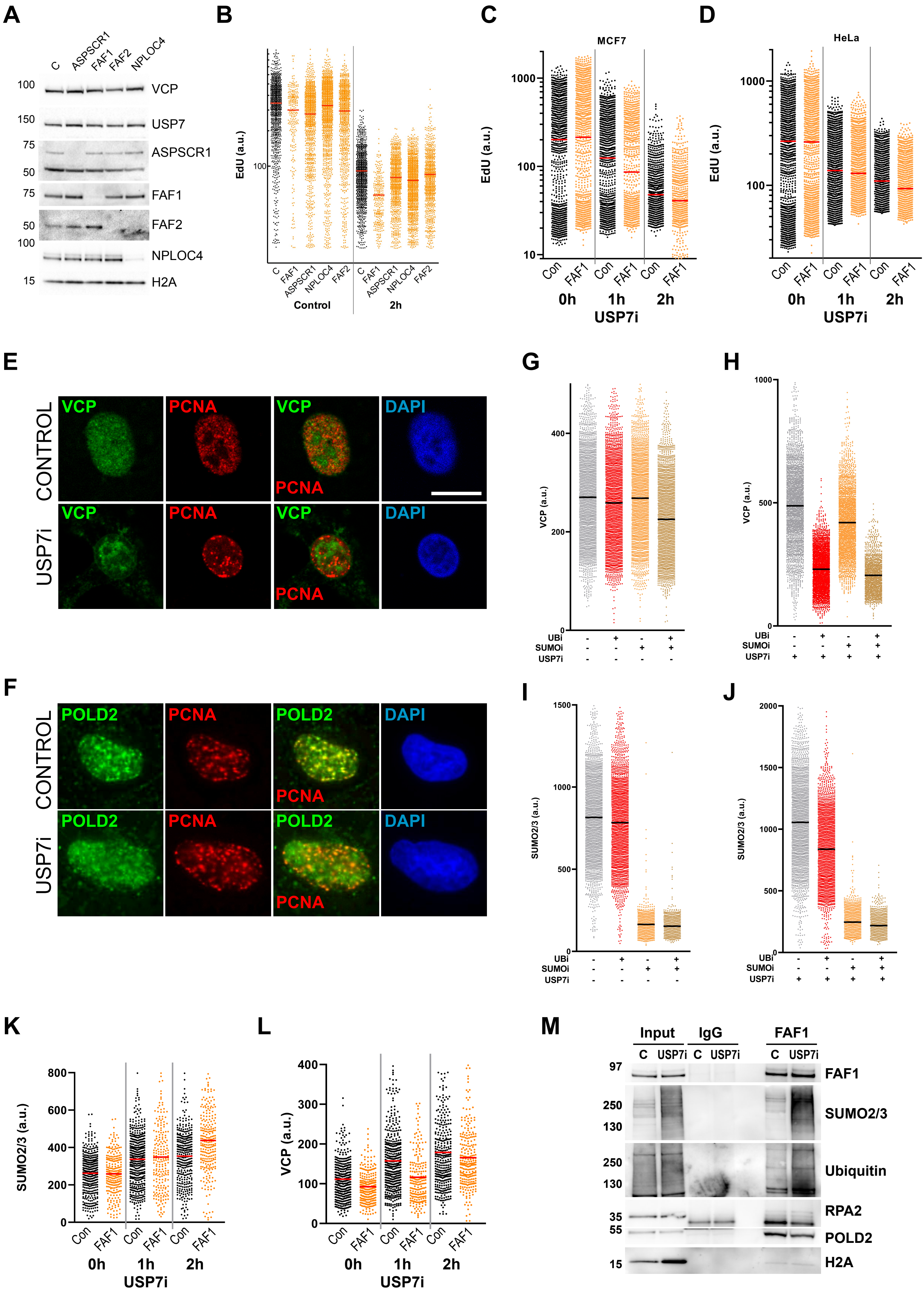

**Supplementary Figure 4. Effect of VCP adaptors during DNA replication.** Related to Figures 3 and 4. (A) RPE cells were transfected with a non-specific siRNA (C) or siRNA pools against different VCP adaptors. The expression of VCP, USP7, and the VCP adaptors was analyzed by Western blot in whole cell extracts 48 h after transfection. The levels of histone H2A are shown as a control. (B) Analysis of the incorporation of EdU by high throughput microscopy in RPE cells 48 h after transfection with a non-specific siRNA (black, C) or a pool of siRNA directed against different VCP adaptors (orange). Cells were treated with DMSO (control) or 10 μM P22077 (USP7i) for 2 h. The experiments were repeated 3 times and one representative result is shown. (C-D) MCF7 (C) and HeLa (D) cells were transfected with a non-specific siRNA (Con, black) or siRNA against FAF1 (FAF1, orange) and the levels of EdU incorporation were measured by high throughput microscopy 48 hrs after transfection. Cells were treated with DMSO (Control) or 25 μM P22077 (USP7i) for the indicated times. (A-D) Related to Figure 3. (E-F) Immunofluorescence analysis of chromatin-bound VCP (green) and PCNA (red) (A), POLD2 (green) and PCNA (red) (B) levels in U2OS cells that were either untreated (CONTROL), or upon treatment with 50 μM USP7i for 4 h. DNA was stained with DAPI (blue). The overlay for the different staining is also shown. Scale bar, 10 μm. (G) Analysis of chromatin bound VCP level by immunofluorescence and high throughput microscopy in U2OS cells synchronized with 1 mM thymidine for 16 h, released for 2 h and then treated with DMSO, 1 μM MLN7423 (Ubi), 2 μM ML792 (SUMOi), or a combination of both for 1 h (Ubi+SUMOi). In (H) cells were subsequently treated with 25 μM P22077 for 2 h in presence of ubiquitin and SUMO inhibitors. (I-J) Analysis of chromatin bound SUMO2/3 levels by immunofluorescence and high throughput microscopy

in U2OS cells treated as in (G-H). (K-L) MCF7 cells were transfected with a non-specific siRNA (Con, black) or siRNA against FAF1 (FAF1, orange) and the level of SUMO2/3 (C) or VCP (D) on chromatin were measured by high throughput microscopy 48 hrs after transfection. Cells were treated with DMSO (Control) or 25 μM P22077 (USP7i) for the indicated times. This experiment was repeated twice with similar results. (M) WB analysis of the immunoprecipitation of FAF1 in nuclear extracts from HCT116 cells treated with DMSO (C) or 50 μM P22077 (USP7i) for 4 h. 1% of the input material is shown together with the pull-down using a non-specific IgG or an antibody specific for FAF1. Specific antibodies against FAF1, SUMO2/3, ubiquitin, RPA2, POLD2, and H2A were used for detection. (E-M) Related to Figure 4A-E.

**Supplementary Figure 5**

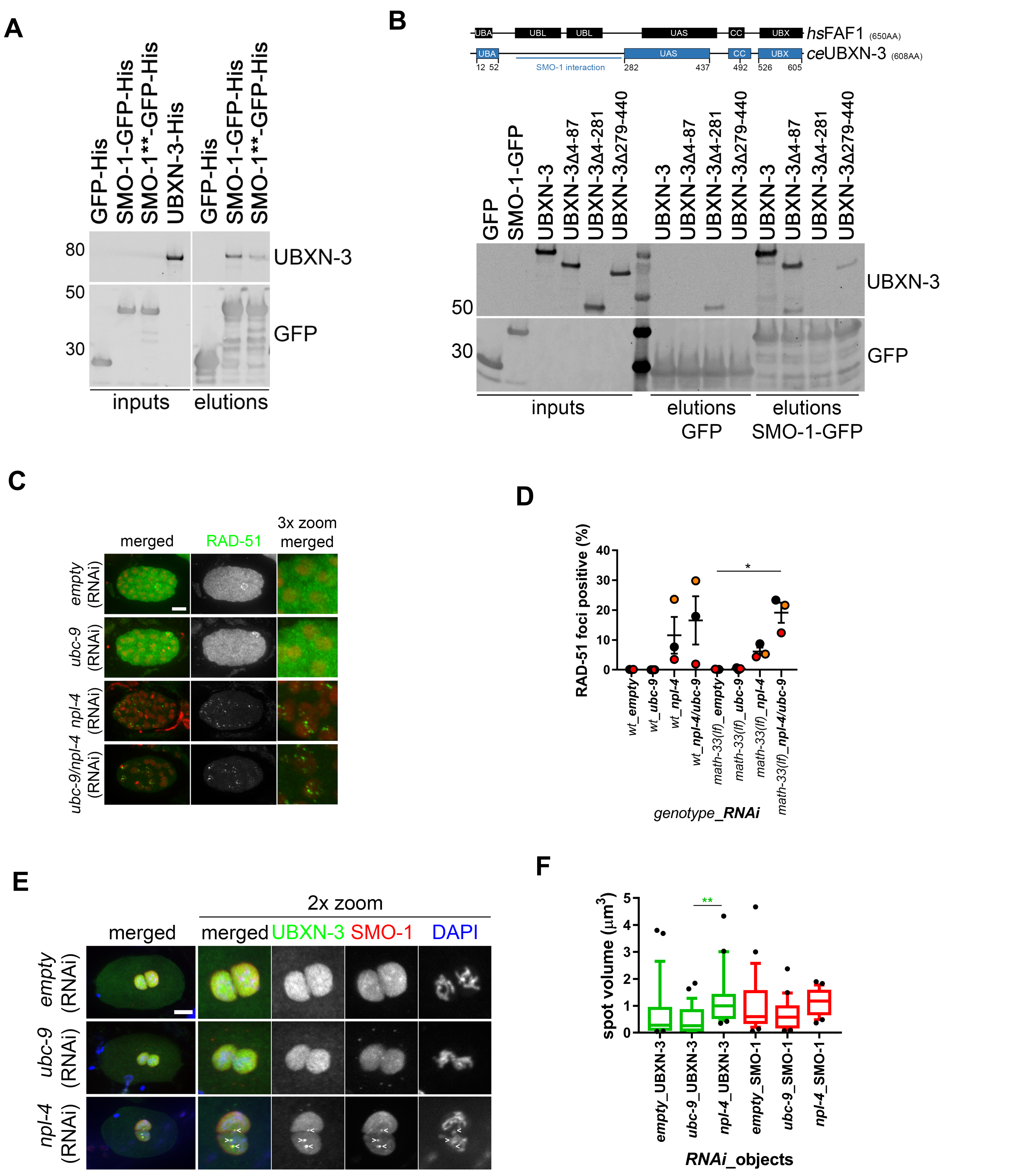

**Supplementary Figure 5. USP7 and VCP$^{FAF1}$ control SUMOylated and ubiquitylated proteins during DNA replication.** Related to Figure 4G-I. (A) Western blot analysis of input and elution fractions of *in vitro* interaction analysis of indicated SMO-1-GFP and SIM binding deficient SMO-1**-GFP proteins with UBXN-3. GFP served as a negative control. (B) Schematic overview of the domain structure of human (*hs*) FAF1 (black boxes) and *C. elegans* (*ce*) UBXN-3b (blue boxes). The position of the displayed domains in UBXN-3 refers to the amino acid (AA) sequence. The region required for SMO-1 interaction is marked for UBXN-3. Western blot analysis of inputs and eluates using respective domain truncations of the UBXN-3b protein (UBXN-3ΔAA). (C) Representative micrographs of wt embryos depleted for indicated RNAi targets. Images show maximum projections of confocal optical sections. Immunostaining was performed for RAD-51 (green) protein together with DAPI (red). (D) Graph shows quantification of embryos displaying nuclear RAD-51 foci in indicated genotypes depleted for respective RNAi targets. Graph shows mean values of three individual experiments, each considering at least 500 embryos per condition. The colors of individual data points indicate matched data in individual experiments. Error bars show standard error of the mean. Asterisks indicate statistical significance in One-way Anova Sidak's multiple comparison test. (*p-value <0,05). (E) Representative micrographs of wt *C. elegans* embryos depleted for indicated RNAi targets. Images show maximum projections of confocal optical sections. Immunostaining was performed for UBXN-3 (green) and SMO-1 (red) proteins as well as DAPI (blue). Arrowheads highlight UBXN-3 nuclear bodies, co-localized with SMO-1 puncta upon *npl-4*(RNAi) depletion. (F) Quantification of UBXN-3 (green) and SMO-1 (red) spot volume in embryos depleted for indicated

gene products. Whisker’s plots of the 10-90 percentile, statistical significance was interrogated using One way Anova Sidak’s multiple comparison test and is indicated by asterisks, (** p-value <0,01). Scale bars represent 10 μm.
